# Object but no room tilt illusion due to apperceptive visual agnosia in a right parieto-occipital lesion disconnecting visual–spatial integration

**DOI:** 10.1007/s00415-025-13260-4

**Published:** 2025-07-21

**Authors:** Philipp J. Koch, Tabea Kürten, Anja Fellbrich, Andreas Sprenger, Christoph Helmchen

**Affiliations:** 1 Department of Neurology, University of Lübeck, University Hospital Schleswig-Holstein, Campus Lübeck, 23538 Lübeck, Germany; 2 Center of Brain, Behavior and Metabolism (CBBM), University of Lübeck, Lübeck, Germany; 3 Department of Neurology and Experimental Neurology, Charité – Universitätsmedizin Berlin, Berlin, Germany; 4 Institute of Psychology II, University of Lübeck, Lübeck, Germany

Dear Sirs,

Room tilt illusion (RTI) is a usually transient disorder of visuo-spatial perception: the entire visual scene is paroxysmally tilted by 90° or 180° (upside down). It is suspected to be linked to a temporary mismatch of visual and vestibular three-dimensional spatial coordinate maps, usually caused by an acute vestibular tone imbalance. The paroxysmal nature may be caused by temporary dominance of either the visual or the vestibular system to maintain spatial orientation using reciprocal inhibitory interaction. Accordingly, most of the lesions affected central or peripheral vestibular structures, particularly the vestibulo-cerebellum [1, 2], the parieto-occipital or frontal cortex, however, sparing the primary visual cortex [3]. While RTI by vestibular lesions usually occurs without alterations of object’s color, shape or size [1, 4], the optic illusion in visual metamorphopsia alters the size, shape, or the angulation of objects [3].

Here, we describe a patient with severe apperceptive visual agnosia with object metamorphopsia and object tilt illusions (OTI) who suffered from paroxysmal 180° tilt perceptions of objects within a stable visual environment, i.e., in the absence of RTI. This was caused by a right-sided posterior reversible encephalopathy syndrome (PRES) inflicting primary and secondary visual cortex and the dorsal part of the superior parietal lobe but sparing core multisensory brain areas processing vestibular signals, particularly the posterior insula/operculum.

The 56-year-old patient developed acute visual symptoms while driving a car: the stationary objects of the visual scene in front of him were moving like “lava” from the right-hand side to the left visual field for a few minutes. Later on, he noticed distortion of visual objects with respect to shape, size and color: the hairs of care takers in the emergency room were perceived as uniform black and their bodies, particularly faces (prosopmetamorphopsia) and heads, appeared squeezed and elongated. Objects in the room were perceived distorted with abnormal size and shape. He also experienced 180° tilts of objects for seconds to minutes, e.g., a distant monitor or even persons in front of him, while there were no OTI within his peripersonal space. This did not change with gaze or head position. Noticeably, his own body perception and the visual reference frame remained stable during these episodes and he did not notice an own involuntary body motion perception (no egomotion). He experienced visual distortion in his own personal space: his hands were different in size and form, even in the intact visual field. Apperceptive visual object agnosia became impressively evident when he tried to put on his black shirt; he visually recognized the collar and its sleeve, confirmed it by tactile exploration but was unable to compute and integrate it to a comprehensive picture of the whole shirt: he repetitively turned it around but failed to properly dress. He also recognized imagined or observed visual targets in his visual field multiple times, for several minutes, irrespective of gaze or head position.

On clinical examination, there was an extensive homonymous hemianopia of the left visual hemifield. Saccades and smooth pursuit eye movements into the left hemianopic field were hypometric and cogwheel, respectively. There were neither clinical signs of ocular apraxia, optic ataxia, alexia, dyscalculia, agnosia for letters (reading only confounded by hemianopia), right–left disorientation in personal and extrapersonal space or multimodal neglect (normal paper pencil test) nor vestibular impairment (spontaneous nystagmus, head impulse test, skew deviation, and gaze-evoked nystagmus). Reading and writing were normal. Speech was fluent and comprehension of spoken or written language intact. Self-paced graphical reconstruction and recall of visual objects were adequate but usually oversized. Facial recognition was normal but perceived distorted. Otherwise, neurological examination was normal.

The patient gave written informed consent for the following examinations. Quantitative visual perimetry revealed macula sparing left homonymous hemianopia. The naming of objects (CERAD) was normal, he recognized 15 of 15 images correctly. The visual object and space perception battery (VOSP) revealed severe deficits in object or space perception (12 from 20 items) when targets were presented in the intact right visual hemifield indicating severe simultaneous agnosia (apperceptive visual agnosia). Accordingly, he had an increased error rate on recognizing Poppenreuther objects (1 from 4 items). Rod and frame test revealed no tilt perception (2° to the left). Quantitative vestibular tests were normal (subjective visual vertical, video head impulse test: gain of the vestibulo-ocular reflex 0.9 bilaterally). The gain of smooth pursuit eye movements (Eyelink 1000 plus, 1000 Hz sampling rate, SR Research, Ottawa/Canada) during predictive pursuit (sinusoidal movement ± 15° at 0.3 Hz) was abnormal in the acute stage (0.72) and recovered within 1 week (gain: 0.95) and abnormal to the left side in non-predictive pursuit (step-ramp, 16°/s, gain: 0.59), whereas movements to the right were nearly in the normal range (gain: 0.81). Visual motion coherence recognition was abnormal at symptom onset on a trend level (first recording after 1 week: 21.7%; t = 1.928, *p* = 0.064) and recovered after 3 months (15.8%; *t* = 0.778, *p* = 0.443), compared with healthy participants [(*N* = 28): mean 11.81% ± 5.04)].

Mental rotation tasks of human bodies (Blender™ Version 2.78; https://www.blender.org) seen from the front or the back, tilted or upside down with either arm extended (Fig. 1, Supplementary data) revealed abnormally increased error rates of the patient (37% error rate, Fig. 1) which recovered after 3 months suggesting abnormal mental rotation transformation in the acute stage. Saccade latency of saccades to the left was increased. MRI (Fig. 1) showed a right-sided (parieto)-occipital lesion involving multiple areas of the visual cortex, the dorsal rim of the superior parietal lobe and the dorsal part of the superior temporal gyrus but sparing multisensory vestibular brain areas, specifically the opercular/posterior insula. Lesions were integrated with normative connectome data to infer an individual disconnectivity profile [5] (see supplementary data for a detailed methodological description). This revealed a disconnectivity fingerprint beyond the lesioned occipito-parietal cortex (Fig. 2): disconnected white matter fascicles were found targeting the ipsilesional right superior parietal lobe (Area 7), superior temporal gyrus (STG), visual motion area V5/MT, medial occipital lobe (cuneus), the angular gyrus in the inferior parietal lobe (Area 39, 40; IPL) and impressively in the splenium and contralateral occipital extrastriate cortex**.** Posterior insula, particularly the operculum (i.e., OP2, the core vestibular region), was spared. Susceptibility-weighted imaging (SWI) disclosed numerous parieto-occipital microbleeds suggesting amyloid angiopathy. Etiologically, the edema was caused by progressive reversible encephalopathy syndrome related to severe hypertension. Cerebral digital angiography was normal, electroencephalography did not show increased brain excitability. Follow-up examination of all tests 3 months later revealed almost complete (except for visual field defects) reversal of symptoms (OTI), signs and MRI lesions, making the presented lesions and disconnections a likely cause of OTI.

**Fig. 1:**
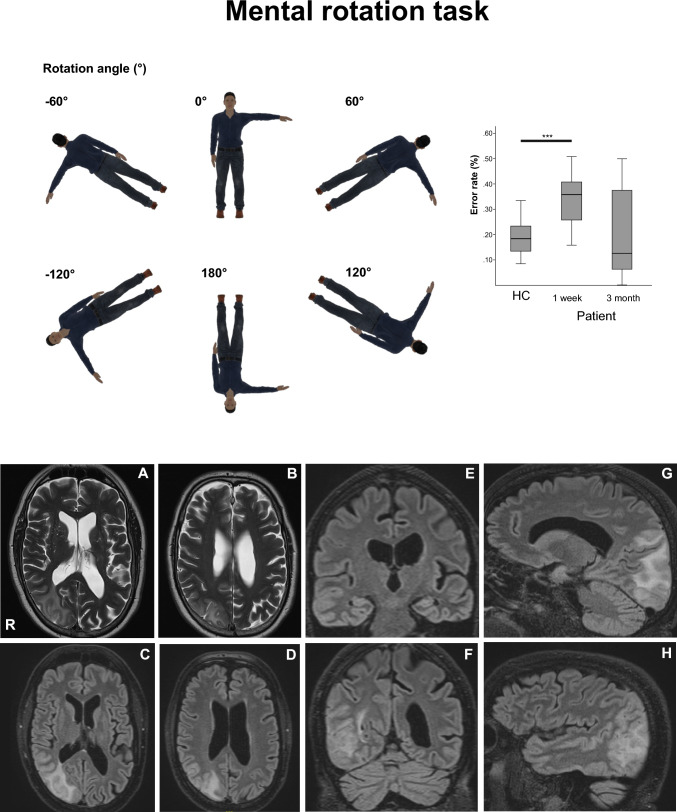
Mental rotation task: The different body positions in space are shown in the upper part with rotation angles (0–60–120–180°, 0 = upright, 180° = upside down body position) as the participant viewed on the monitor. The patient’s higher mean error rate (% ± SEM) of the mental rotation task 1 week after symptom onset and its recovery after 3 months is displayed in the bar plots compared to healthy control subjects (**** = *p* < 0.001). The lower part shows the MRI lesion spread of edema. The right occipital lesion (PRES) is shown in axial (**A, B** with T2 TSE und **C, D** with T2 space dark fluid sequences), coronal (**E, F**) and sagittal (**G, H**) slices. The operculum and right posterior insula (**A, C, E**) and the intraparietal sulcus (**F**) are spared by the edema. PRES resolved completely after 3 months in a follow-up MRI imaging session using the same sequences

**Fig. 2:**
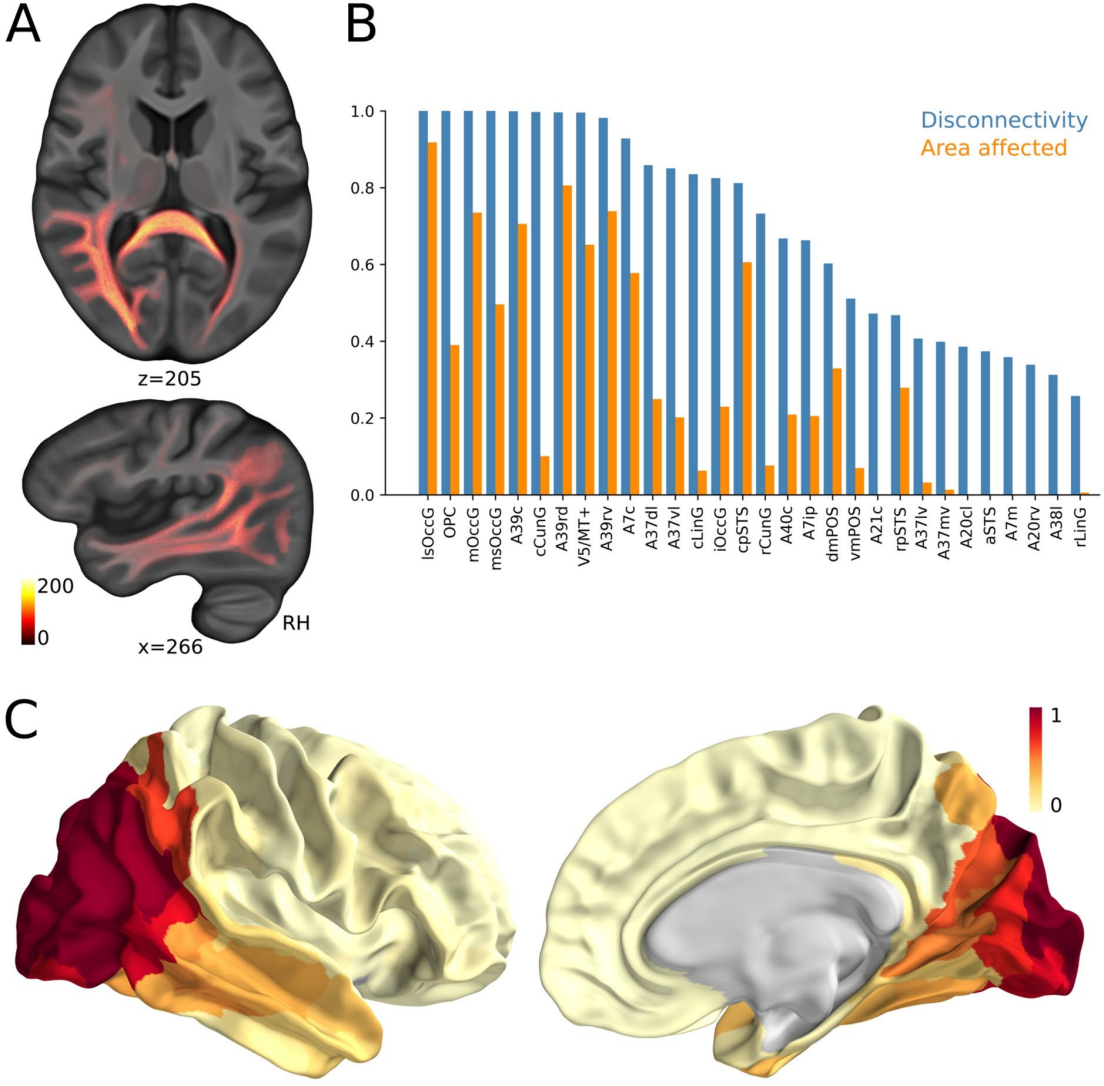
Disconnectivity analysis. An average tractogram was used to identify streamlines (SL) that spatially intersected the manually defined lesion mask. These intersecting streamlines were classified as disconnected. **A** All disconnected streamlines were voxelwise projected onto the HCPA422 T1-weighted template, with voxel color intensity reflecting the number of disconnected streamlines passing through each voxel, i.e., tract density imaging. *Z-* and *X*-axes are given. **B** Using the Brainnetome atlas, (i) the proportion of each cortical area affected by the lesion (orange) and (ii) the proportion of streamlines disconnected from each cortical region (blue) were quantified. 1 indicates that the whole volume of the cortical area is covered by the lesion, or all SL connected to the particular area are disconnected. Only the top 30 disconnected areas are displayed, all located in the right hemisphere. **C** Percentage of disconnected streamlines per region is visualized on the cortical surface of the right hemisphere. For abbreviations, see supplementary data

This case is remarkable for at least two reasons: (i) it describes paroxysmal OTI without room tilt perception, i.e., within a stable personal and extrapersonal visual space, in the absence of immediate lesions or disconnections of the multisensory core vestibular areas in the opercular cortex and (ii) disconnectivity analysis disclosed profound occipital–parietal visual–spatial disconnections reaching beyond the lesion edema (bar plot in Fig. 2B) which probably provoked a temporary mismatch of visual–spatial reference maps.

RTI is suspected to be linked to a temporary mismatch of visual and vestibular three-dimensional spatial coordinate maps, usually caused by an acute vestibular tone imbalance. In contrast to our patient, brain lesions in RTI usually affected central or peripheral vestibular structures. RTI have not been associated with unilateral occipital lesions yet, neither in those with reversal visual metamorphopsia [3] nor with unilateral PRES. This patient ‘s OTI is probably related to his gross apperceptive visual agnosia, i.e., the patient probably did not perceive an RTI, since he could not spatially compute distinctly separate visual objects to a coherent comprehensive percept of the visual surroundings. The abnormally moving visual surrounding from the intact to the hemianopic visual field at symptom onset may be related to edema spreading to the right MT/V5 at disease onset or the posterior rim of the human homologue of the dorsal medial superior temporal area (MSTd) which is in line with the moderately impaired smooth pursuit eye movements and the still measurable trend to abnormal normal motion coherence perception 1 week after symptom onset. MSTd encodes optic flow in head and eye-centered reference frames. The patient’s lesion spared the ventral intraparietal area (VIP) in the intraparietal sulcus, encoding movements in space-centered coordinate frames, but it was considerably disconnected (A40c; Fig. 2). VIP and MST neurons can process extraretinal information of eye movement and self-motion in order to estimate heading even from distorted flow fields, whereas VIP neurons further analyze the structure of the retinal motion patterns to maintain heading selectivity irrespective of eye movement [6]. The neuronal tuning for visual and vestibular headings are opposite rather than aligned [7], making switches between both coordinate frames in case of lesions or disconnections feasible. The edema elicited disconnections of intrahemispheric connections between VIP and MST with consecutive transient misalignment of eye/head and space-centered reference frames. Moreover, unilateral cerebral lesions generate an imbalance of optic flow processing, resulting in ipsiversive visual distortions [6], e.g., the moving roadside on the right-hand side of our patient with right-hemispheric edema at symptom onset.

Noticeably, the patient showed an abnormally high error rate in the mental rotation transformation task [8]. Mental rotation transformation of bodies [9] and objects requires intact bilateral parietal and temporo-occipital function. Visuomotor control and visuospatial processing providing self-motion and object-motion perception by using egocentric coordinates and viewer-centered reference frames are largely mediated by the dorsal visual stream. It plays a crucial role in identifying the spatial relation of an object’s components and projects to the ventral visual stream, thereby contributing to object and shape recognition. This impairment of recognizing part relations might be a plausible explanation for the patient’s inability to recognize the composite function of visible elements (sleeve and collar) of his t-shirt. It is in accord with patients with right parietal lesions with particular agnosia for composite object orientation [10]. The dorsal visual pathway originates in the primary visual cortex and terminates in the superior parietal lobule (SPL), which was lesioned by our patient’s edema. This is in accord with the patient’s difficulties in localizing objects in space. This may result in object tilt perceptions if reference frames of visual perception are changed. The ventral visual pathway, projecting to the temporal lobe, was functionally affected due to the gross misperception of object shapes (visual metamorphopsia) and colours. It was only dorsally lesioned but clearly disconnected. The discrimination of rotated stimuli has been linked to the lateral part of the posterior superior temporal gyrus and the mesial part of the inferior parietal lobule [11].

Damage to the SPL may result in apperceptive visual agnosia, i.e., simultanagnosia, as it computes the integration of multiple visual components. Whereas simultanagnosia is usually described in patients with Balint syndrome resulting from bi-hemispheric parieto-occipital lesions inflicting the SPL, several reports suggest that even unilateral lesions may cause simultanagnosia.

The superior and inferior parietal lobules are activated using fMRI during mental rotation tasks concerning two- and three-dimensional objects [12]. They are implicated in visuospatial transformation and responds to tasks on mental rotation and mirroring, orientation of surfaces and discrimination of angles and in the processing of the contextual cues that contribute to our perception of egocentric space. Remarkably, the BOLD signal in the SPL grows stronger upon increasing the rotation angle, reflecting a quantitative and linear relationship between neuronal activation and task difficulty [12]. Furthermore, a right-hemispheric dominance in specialization on mental rotation tasks has been suggested in several studies [12].

Noticeably, the gross disconnection in the splenium (Fig. 2A) leads to a functional bilateral lesion facilitating simultanagnosia as integration of visual information is interhemispherically integrated via fibers of the posterior splenium [13]. The disruption of callosal fibres of this bilateral network may cause an asymmetry in inter-hemispheric visuo-spatial orientation causing mental rotation impairment [14]. Evidence comes from callosotomized patients who were unable to perform mental rotation [14].

Abnormal error rates in mental egocentric rotation task may also indicate a visuo-vestibular coordinate mismatch as it is impaired during vestibular stimulation [8]. There was no evidence for abnormal visual–vestibular interaction coming from clinical or quantitative vestibular tests nor from the edema morphology; core structures of vestibular processing, i.e., the operculum (OP1–4), in particular the posterior insula important for self-motion perception, including the visual area of the cingulate (CSv) were spared, neither by edema nor by disconnected streamlines. In accord, the patient did not notice abnormal motion of the own body often found with vestibular lesions nor was the tilted object modified by head position. In contrast to temporo-parietal junction lesions, our patient did not report altered embodiment or body ownership. Strikingly, apart from occipital regions the strongest disconnected areas were those known to be involved in mental rotation tasks: the IPL with the angular gyrus (A39, A40), A7 (SPL), and V5 (MT)[15]. This disconnectivity possibly accounts for the patient’s striking visuo-spatial impairments.

The paroxysmal nature of this OTI may be caused by the initial edema or microbleeds disclosed in SWI in the right parieto-occipital cortex suggesting comorbid cerebral amyloid angiopathy.

In summary, this case study illustrates object tilt illusions without room tilt illusions which may be related to impairments of reference frames of visual perception as we did not find evidence by clinical, behavioral and brain imaging analyses that support lesions or disconnections of core vestibular processing areas as it is postulated in RTI. As perceived tilts were only found in distant but not near objects and noticed without any tilt perception of one’ own body a visual illusion within the extrapersonal space might be a feasible mechanism [3] facilitated by the inter- and intrahemispheric occipito-parietal disconnections demonstrated by modern disconnectivity analyses. As a consequence, information on the visual representation of objects in space may not properly be matched between both hemispheres (due to the severe disconnection in the splenium) leading not only to object distorsions and metamorphopsia but also OTI.

## Supplementary Information

Below is the link to the electronic supplementary material.

Supplementary file1 (DOCX 38 KB)

## Data Availability

The data that support the findings of this study are available from the corresponding author, upon reasonable request.
