# Supplementary material for: Object but no room tilt illusion due to apperceptive visual agnosia in a right parieto-occipital lesion disconnecting visual–spatial integration
Source: J Neurol. 2025 Jul 21;272(8):527. doi: 10.1007/s00415-025-13260-4 (PMC12279592; doi:10.1007/s00415-025-13260-4)
Supplement: Supplementary file 1 — Supplementary file1 (DOCX 38 KB) [file 415_2025_13260_MOESM1_ESM.docx]

# Methods:

# Mental rotation task: Images of human bodies (created by using Blender^TM^ Version 2.78; <https://www.blender.org>) were presented on a monitor screen: tilted at 0° (upright), 60°, 120°, 180° (upside down), -60°, and -120°, with either arm extended and viewed from the front or the back (48 trials). The participant was sitting with the head positioned on a chin rest in an otherwise darkened room, without other visual cues. The participant was instructed to indicate which arm of the presented body is lifted by imagining one’s own body rotation to achieve the target body position by pressing a response button with the index finger as fast as possible. The interval of the target presentation (duration maximal 4 sec) to the response on the button was recorded and the number of errors were analysed.

# Disconnectivity analysis: Disconnectivity analyses were performed based on clinical lesion information, following a similar approach as described previously (Frontzkowski *et al.*, 2024). The edema lesion was manually delineated by PJK using mrview from MRtrix3 (https://www.mrtrix.org) based on a T2-weighted fluid-attenuated inversion recovery (FLAIR) sequence (TR = 9000 ms, TE = 0.114 ms, TI = 2500 ms, flip angle = 180°, 28 transverse slices, voxel size = 0.5 × 0.5 × 5.5 mm³). The lesion mask was coregistered to the HCPA422 T1-weighted template (0.5 × 0.5 × 0.5 mm³) using an affine and deformable transformation with mutual information as the cost function, implemented in Advanced Normalization Tools (ANTs; <https://github.com/ANTsX>). For disconnectivity analyses, an average tractogram derived from 422 HCP-Aging subjects, containing two million streamlines (SL) (Xiao *et al.*, 2023), was used. Streamlines spatially intersecting the lesion were marked as disconnected and mapped voxel-wise to generate tract-density images (Figure 2 A). Region-of-interest (ROI) analyses were conducted using the Brainnetome atlas (https://doi.org/10.1093/cercor/bhw157). First, the volumetric proportion of each cortical area affected by the lesion was calculated. Second, the proportion of streamlines disconnected from each cortical region was quantified (Figure 2 B,C).

# Abbreviations (Fig.2): A20cl: Caudolateral Area 20, Inferior Temporal Gyrus; A20rv: Rostroventral Area 20, Inferior Temporal Gyrus; A21c: Caudal Area 21, Middle Temporal Gyrus; A37dl: Dorsolateral Area 37, Fusiform Gyrus; A37lv: Lateroventral Area 37, Fusiform Gyrus; A37mv: Mediolateral Ventral Area 37, Fusiform Gyrus; A37vl: Ventrolateral Area 37, Fusiform Gyrus; A38l: Lateral Area 38, Superior Temporal Pole; A39c: Caudal Area 39, Inferior Parietal Lobule; A39rd: Rostrodorsal Area 39, Inferior Parietal Lobule; A39rv: Rostroventral Area 39, Inferior Parietal Lobule; A40c: Caudal Area 40, Inferior Parietal Lobule; A7c: Caudal Area 7, Superior Parietal Lobule; A7ip: Intraparietal Area 7, Superior Parietal Lobule; A7m: Medial Area 7, Superior Parietal Lobule; aSTS: Anterior Superior Temporal Sulcus; cCunG: Caudal Cuneus Gyrus; cLinG: Caudal Lingual Gyrus; cpSTS: Caudal part of the Superior Temporal Sulcus; dmPOS: Dorsomedial Parieto-Occipital Sulcus; iOccG: Inferior Occipital Gyrus; IsOccG: Isthmus of the Occipital Gyrus; mOccG: Medial Occipital Gyrus; msOccG: Middle Superior Occipital Gyrus; OPC: Occipital Pole Cortex; rCunG: Rostral Cuneus Gyrus; RH: right hemisphere; rLinG: Rostral Lingual Gyrus; rpSTS: Rostral part of the Superior Temporal Sulcus; V5/MT+: Visual Area V5 / Middle Temporal Complex; vmPOS: Ventromedial Parieto-Occipital Sulcus

#

# References:

Frontzkowski L, Fehring F, Frey BM, Wrobel PP, Reibelt A, Higgen F*, et al.* Frontoparietal Structural Network Disconnections Correlate With Outcome After a Severe Stroke. Hum Brain Mapp 2024; 45(16): e70060.

Xiao Y, Gilmore G, Kai J, Lau JC, Peters T, Khan AR. A population-averaged structural connectomic brain atlas dataset from 422 HCP-aging subjects. Data Brief 2023; 50: 109513.
